# Analog Resistive Switching and Artificial Synaptic Behavior of ITO/WO_X_/TaN Memristors

**DOI:** 10.3390/ma16041687

**Published:** 2023-02-17

**Authors:** Youngboo Cho, Jihyung Kim, Myounggon Kang, Sungjun Kim

**Affiliations:** 1 Division of Electronics and Electrical Engineering, Dongguk University, Seoul 04620, Republic of Korea; 2 Department of Electronics Engineering, Korea National University of Transportation, Chungju-si 27469, Republic of Korea

**Keywords:** memristor, reactive sputtering, tungsten oxide, artificial synaptic devices, neuromorphic computing

## Abstract

In this work, we fabricated an ITO/WO_X_/TaN memristor device by reactive sputtering to investigate resistive switching and conduct analog resistive switching to implement artificial synaptic devices. The device showed good pulse endurance (10^4^ cycles), a high on/off ratio (>10), and long retention (>10^4^ s) at room temperature. The conduction mechanism could be explained by Schottky emission conduction. Further, the resistive switching characteristics were performed by additional pulse-signal-based experiments for more practical operation. Lastly, the potentiation/depression characteristics were examined for 10 cycles. The results thus indicate that the WO_X_-based devices are appropriate candidates for synaptic devices as well as next-generation nonvolatile memory.

## 1. Introduction

Nonvolatile memory (NVM) devices with high switching speeds and high-density integration should be developed for the effective implementation of artificial intelligence (AI) technologies [1]. Because of the low cost and high compatibility of flash memory for complementary metal-oxide-semiconductor (CMOS) fabrication, silicon-based flash memories are the current market leaders for NVMs [2]. However, owing to physical limitations, we have reached the limit for increasing the memory density [3]. Accordingly, several candidates have emerged as replacements for traditional memory for NVM devices, including the ferroelectric field-effect transistor (FeFET) [4,5], phase-change memory (PCM) [6], magnetic random-access memory (MRAM) [7], and resistive random-access memory (RRAM) [8]. Among these emerging memories, RRAM has shown great potential for its advantages, such as fast and low power switching, high endurance, multilevel characteristics, applicability to flexible memory [9], and a high on/off ratio [10,11,12,13,14]; moreover, it has excellent scalability owing to its simple metal–insulator–metal (MIM) structure [15]. This scalability property of RRAM is one of the many reasons it has been widely studied for high-density cross-point arrays [16] and three-dimensional vertical RRAM (3D VRRAM) [17]. In addition, RRAM devices can be used as artificial synaptic devices for neuromorphic computing because of the advantages described above [18,19,20,21]. Neuromorphic systems are emerging as alternatives to the Von Neumann structure, which is the existing computing method, because it has reached its limit due to bottlenecks [22]. Neuromorphic computing mimics the behavior of the human brain [23] and requires an artificial cell that acts as a synapse, for which the RRAM cell may be an appropriate candidate [18].

Digital information is stored in the form of binary numbers “0” or “1”. In the case of RRAM, the stored information is determined to be “0” or “1” based on the resistance state [24], where the low-resistance state (LRS) corresponds to “1” and the high-resistance state (HRS) corresponds to “0”. RRAM devices are usually in the HRS initially [25]. To switch the device from the initial state, a procedure called “forming” is required; forming refers to the application of high-voltage stress that causes a soft breakdown in the insulator layer. In addition, a compliance current (CC) should be applied to protect the switching layer from permanent breakdown when applying the forming voltage [26]. After changing to the LRS, a “reset” procedure that applies a voltage of opposite polarity to the “forming” voltage is required to return to the HRS. To switch to LRS from HRS again, a “set” procedure is needed; the “set” process involves applying a voltage of opposite polarity to the “reset” voltage. The range of current flow between the LRS and HRS as well as the voltage range required for “set” and “reset” vary from one device to another [25]; hence, these can be used as criteria to evaluate the power consumption of an RRAM device [27].

Presently, researchers are investigating suitable materials to realize more ideal RRAM characteristics. Resistive switching has been observed in transition-metal oxides (TMO), such as zinc, tantalum, and tungsten oxides [28,29,30]. In particular, tungsten oxide is a compatible material with CMOS technology [31], making it easier to integrate RRAM into the existing back-end-of-line Si technologies. Each TMO used in the switching layer exhibits various characteristics. However, the type of TMO used affects the switching characteristics of the device, in addition to the composition ratio of the metal oxide and the number of oxygen vacancies within a given material [32]. Resistance switching in TMO systems is often based on the formation and rupture of the conductive filament (CF) that usually comprises oxygen vacancies [33]. The composition ratio of a TMO can be varied by changing the gas flow rate ratio of oxygen (O_2_) and argon (Ar) during sputtering [34]. Moreover, the cost and commercial availability should be considered when choosing materials for the RRAM. In addition, if indium tin oxide (ITO) is selected as an electrode, the device can be employed in light-based applications, such as transparent memory [35], photodetectors [36], and displays, owing to its transparency [37].

In this study, we investigate WO_X_-based memristor devices in which the tungsten oxide as the switching layer is deposited by reactive sputtering.

Electrical experiments are then conducted to characterize the resistive switching of the device. Furthermore, the device is tested for its applicability in the pulsed operation mode and as an artificial synaptic device in neuromorphic engineering.

## 2. Materials and Methods

### 2.1. Preparation Method

The fabrication process of the ITO/WO_X_/TaN RRAM device is as follows: First, a 100 nm TaN bottom electrode (BE) layer was deposited on a SiO_2_/Si substrate by direct current (DC) sputtering using a Ta target (99.95% purity) in a mixture of Ar and N_2_ gases. Then, a ~25 nm WO_X_ thin-film insulator was deposited as a switching layer by radio frequency (RF) sputtering using a W target (99.95% purity) in a mixture of Ar and O_2_. The WO_X_ layer was deposited with an O_2_-gas flow rate of 66.7% {O_2_/(Ar + O_2_) = 20 sccm/(10 + 20) sccm}. Finally, a 100 nm thick ITO top electrode (TE), which was deposited by RF sputtering using an ITO target (99.99% purity) in Ar gas, was defined by a mask aligner process for a cell size of 100 μm × 100 μm.

### 2.2. Test and Characterization

The cross-sectional view and film thickness of the ITO/WO_X_/TaN structure was confirmed using a transmission electron microscope (TEM, KANC, Suwon, Republic of Korea) with focused ion beam (FIB) milling. The electrical characteristics and pulse measurement of the devices were obtained using a semiconductor parameter analyzer (Keithley 4200-SCS and PMU ultrafast mode, Tektronix Inc., Beaverton, OR, USA); an external bias evoked from the analyzer module was applied to all ITO TE devices with the TaN BE being grounded.

## 3. Results and Discussion

**Figure 1:**
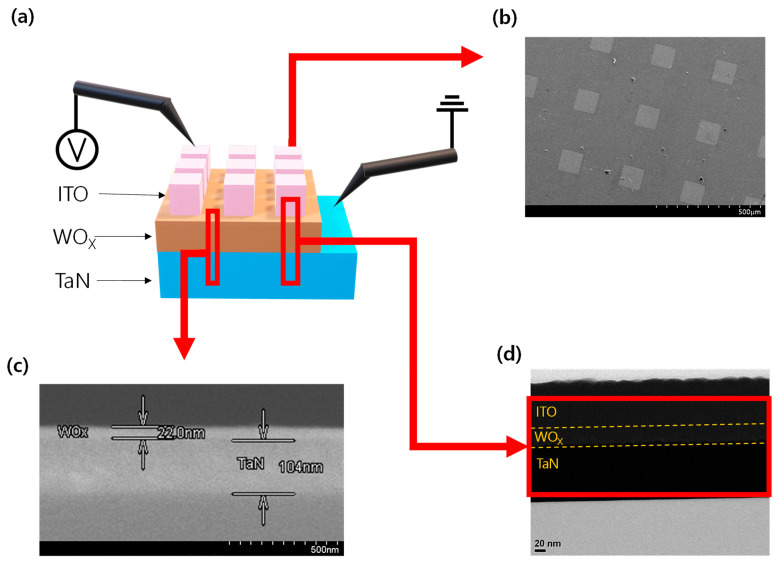
(**a**) Schematic illustration of the ITO/WO_X_/TaN device structure. (**b**) SEM image of the top electrode patterns. (**c**) SEM image of the WO_X_/TaN layers. (**d**) TEM image of ITO/WOx/TaN layers.

Figure 1a shows the schematic of the structure of the ITO/WO_X_/TaN device. A probe tip used to apply the external voltage was placed in contact with the ITO TE, and another probe tip for grounding was placed in contact with the TaN BE. We confirmed the presence of the main layers of the device from the cross-sectional TEM image and SEM image. Figure 1b shows the top-view SEM image of the device. Figure 1c is a cross-sectional SEM image of an area without ITO TE. As shown in Figure 1d, a WO_X_ layer of about 25 nm thickness was deposited between the upper ITO and lower TaN layers; the layers are separated by the yellow dotted lines.

**Figure 2:**
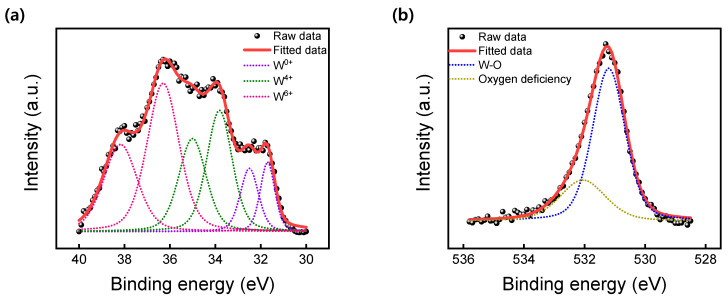
XPS spectra of the WO_X_ switching layer. (**a**) Three different doublets of W 4f core level spectra. (**b**) O 1s core level spectra.

The chemical state of the WO_X_ bulk layer was investigated by X-ray photoelectron spectroscopy (XPS). Figure 2a,b shows the XPS spectrum of W 4f and O 1s, respectively. The binding energy of the pink dotted line at 38.21 eV (W 4f_5/2_) and 36.29 eV (W 4f_7/2_) corresponds to the W^6+^ oxidation state. Likewise, the green and pink doublets correspond to the W^5+^ oxidation state and the W^4+^ oxidation state, respectively. The spectrum of O 1s has doublet peaks, of which one is oxygen deficiency (532.08 eV) and the other is W-O bonding (531.21 eV) [35].

**Figure 3:**
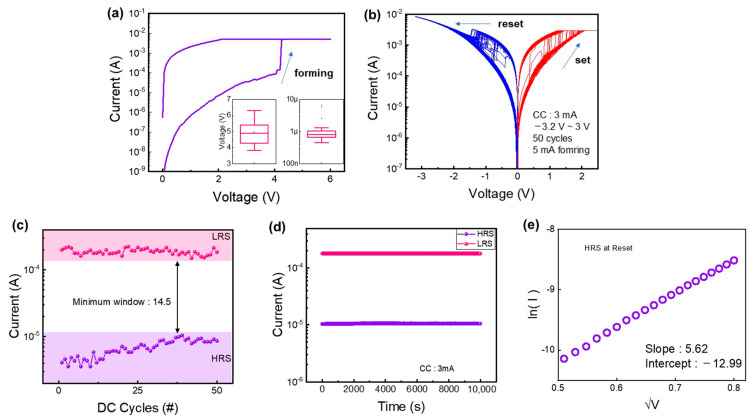
(**a**) Forming voltage statistics based on 45 cells of each of the devices and initial current statistics based on 30 cells before the forming process. (**b**) I–V curves in 50 cycles by DC sweep. (**c**) Endurance and (**d**) retention of the device. (**e**) In(I) versus V^1/2^ for typical I–V curve in HRS.

The electrical properties of the device are described below. First, the forming curve is shown in Figure 3a. The forming voltage was 4.91 V from the measurement of 45 cells and the initial current at the reading of 1 V was 779 nA from 30 cells. It is noted that the initial current was quite high because the sputtering deposited WO_X_ film had a lot of oxygen vacancies. Figure 3b shows the I–V characteristics of the device after the forming process. The DC sweep of 50 cycles was conducted under the same CC (3 mA) and voltage range (−3.2 V to 2.5 V) to achieve repetitive counterclockwise bipolar resistive switching. The current of the device increased when the positive bias was applied for a set process. Conversely, the current decreased with negative bias for a reset process. The possible switching mechanism of the device was the change of the TaON layer [38]. TaN was initially easily oxidized with the WO_X_ layer. The TaON layer was reduced as the oxygen vacancies in WO_X_ migrated to the TaON layer with a positive bias. In other words, as the thickness of TaON decreased, the conductance of the entire memristor system increased. Conversely, the conductance decreased with a negative bias as the TaON layer became thicker.

To investigate the conduction mechanism based on the barrier height modulation of the TaON layer, the I–V curve of the device in the HRS was fitted and interpreted based on the Schottky emission conduction mechanism. Figure 3e shows the fitting plots and Schottky slope values. The result of Schottky current fitting was linear. This indicates that carrier transport was dominated by Schottky emission [39]. Equation (1) is used for the Schottky emission conduction:

(1) $$J=A^{*}T^{2}exp\left[-\frac{q\left(\emptyset_{B}-\sqrt{\frac{qE}{4\pi \varepsilon_{i}d}}\right)}{kT}\right]$$

where $A^{*}$ is the effective Richardson constant, T is the Kelvin temperature, $\emptyset_{B}$ is the Schottky barrier height, $\varepsilon_{i}$ is the dielectric constant, k is the Boltzmann constant, and d is the Schottky barrier distance which is the switching gap of the RRAM. J is the current density and it can be expressed as $\frac{I}{A}$. A is the effective RRAM area, which is the cross-sectional area perpendicular to the direction in which the current flows. To evaluate the $ln\left(\frac{I}{T^{2}}\right)$ vs. $\sqrt{V}$ fitting curve, the Schottky emission conduction formula was transformed into the following equation:

(2) $$ln\left(\frac{I}{T^{2}}\right)=\frac{q\sqrt{\frac{qE}{4\pi \varepsilon_{i}d}}}{kT}\sqrt{V}-\frac{q\emptyset_{B}}{kT}+lnAA^{*}$$

This equation can be considered a linear function of $\sqrt{V}$ and $ln\left(\frac{I}{T^{2}}\right)$. Therefore, we plotted the I–V curves with the *x*-axis as $\sqrt{V}$ and Y axis as $ln\left(\frac{I}{T^{2}}\right)$; here, $\frac{q\sqrt{\frac{qE}{4\pi \varepsilon_{i}d}}}{kT}$ is the slope of the linear function, and $-\frac{q\emptyset_{B}}{kT}+lnA^{*}$ is the intercept. Since the other parameters except $-\emptyset_{B}$ in the intercept are constants, the intercept can be said to be proportional to $-\emptyset_{B}$. Hence, an increase in the intercept indicates a decrease in the Schottky barrier height, which indicates a decrease in the dielectric. Meanwhile, since the other parameters except for $\sqrt{\frac{1}{\varepsilon_{i}d}}$ in the slope are constants, the slope is considered proportional to $\sqrt{\frac{1}{\varepsilon_{i}d}}$. Thus, if the dielectric decreases, the slope should increase.

Figure 3c shows the DC endurances of the devices at a read voltage of −0.1 V. In addition, it is possible to obtain the on/off ratios related to the read margin, which is one of the important characteristics of a memory device [40]. The minimum on/off ratio is calculated by dividing the minimum value among the currents in the LRS by the maximum value among the currents in the HRS. The devices showed no significant decay during 50 repetitions of the DC sweep but had different minimum on/off ratios. The results of the retention test are shown in Figure 3d. The retention test was conducted for 10^4^ s at a reading voltage of −0.1 V at room temperature after experiencing 100 switching cycles. The device did not experience serious degradations under the time conditions.

**Figure 4:**
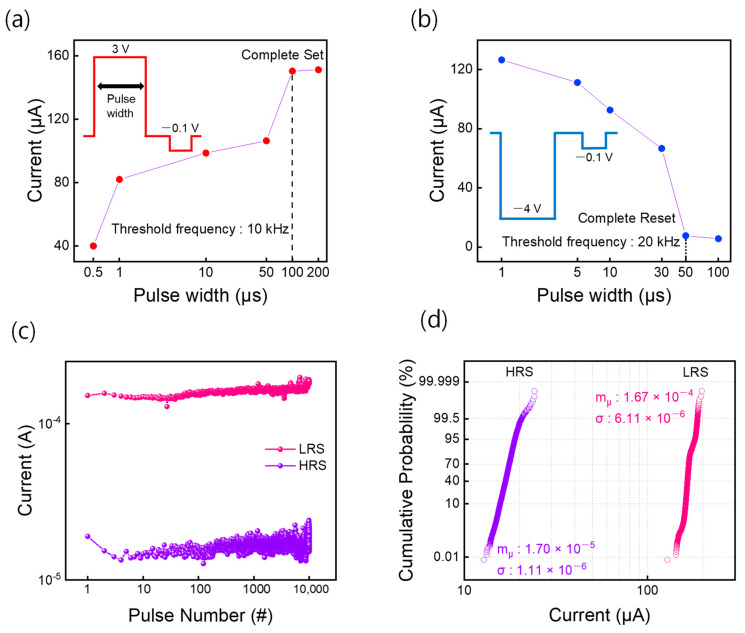
Pulse characteristics of Device #3. (**a**) Current values after applying a single set pulse for different pulse widths. (**b**) Current values after applying a single reset pulse for different pulse widths. (**c**) The pulse endurance test conducted with the optimal set pulse (3 V, 100 μs) and reset pulse (–4 V, 50 μs). (**d**) Cumulative probability obtained from the pulse endurance test.

Additional pulse measurements were performed on the devices to demonstrate the practical operation of the device. For the pulse-switching endurance test, the optimal pulse condition to switch the device was used, as shown in Figure 4a,b. For the same voltage amplitudes (set: 3 V, reset: −4 V), we observed the points at which a complete set or reset occurred. Further, the shortest pulse width that can achieve complete switching (set: 100 μs, reset: 50 μs) was chosen to improve the speed of operation. Pulse endurance tests of 10^4^ cycles were conducted under the pulse conditions in Figure 4c. The device suffered no severe read/write disturbances after more than 10^4^ cycles. For further analysis, the pulse-switching data were compiled into a cumulative probability plot, as shown in Figure 4d. The mean and standard deviation of the current distributions for the HRS and LRS are given in Figure 4d.

**Figure 5:**
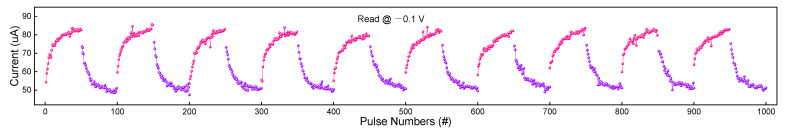
Ten cycles of potentiation (pink) and depression (violet) of the device for 50 identical set pulses (1.25 V, 1 ms) followed by 50 identical reset pulses (−1.40 V, 1 ms) in each cycle.

Finally, we demonstrated 10 cycles of potentiation and depression, as illustrated in Figure 5. The positive pulse amplitude was tuned to 1.25 V and the negative one was tuned to −1.40 V, while the pulse width was set to 1 ms. The read voltage was −0.1 V, which was small enough to not disturb operation. One cycle consisted of 50 identical set pulses followed by a sequence of 50 identical reset pulses. The device showed good reproducibility for 10 cycles. The confirmation of the potentiation and depression characteristics proves that the device can be applied to neuromorphic and artificial synaptic devices. Table 1 illustrates the comparison of electrical parameters and synaptic properties of existing WO_X_-based memristors.

## 4. Conclusions

In conclusion, the WO_X_-based RRAM device successfully mimicked biological synaptic functions for use in neuromorphic computing. Through some important electrical tests, it was determined that all the devices showed stable switching endurances for 50 cycles and lengthy retention above 10^4^ s. Additionally, the device endured 10^4^ switching cycles well with pulsed signals. The potentiation/depression characteristics of the device, which suggests its similarity to the neurotransmitters in biological synapses, were also evaluated. Taking the exploratory results into consideration, we believe that the RRAM device structure proposed in this study is a promising avenue for future research on NVM and neuromorphic devices.

## Figures and Tables

**Table 1:** Comparison of WO_X_-based memristors from previous studies.

Device Structure	Process	Forming Voltage (V)	on/off Ratio	Endurance (#)	Retention (s)	Synaptic Properties	Ref.
Au/WO_3_/FTO	PLD	2.6	10^2^	>10^4^	>10^4^	N/A	[40]
Al/WO_X_/ITO	Sol-gel	Forming-free	~10^3^	>10^2^	N/A	N/A	[41]
TiN/WO_X_/W	RTO	3.5	>10^2^	>10^8^	N/A	N/A	[42]
Al/WO_X_/W	Plasma oxidation	4	10	>40	>10^6^	N/A	[30]
ITO/WO_X_/ITO	Sputtering	Forming-free	~5	300	>10^4^	Potentiation/ Depression	[35]
ITO/WO_X_/TaN	Sputtering	4.91	>10	>10^4^	>10^4^	Potentiation/ Depression	(This work)

## Data Availability

Not applicable.
